# Early- and Long-term Outcomes of Cardiovascular Surgery via Minimal Right Vertical Infra-axillary Thoracotomy: A 15-year Study of 1,126 Patients

**DOI:** 10.1038/s41598-018-22824-6

**Published:** 2018-03-12

**Authors:** Qiang Wang, Jia-Xin Ye, Min Ge, Dong-Jin Wang

**Affiliations:** Department of Thoracic and Cardiovascular Surgery, the Affiliated Drum Tower Hospital of Nanjing University Medical School, Nanjing, 210008 China

## Abstract

This study reviews our results and experience with cardiothoracic surgery via RVIAT over the past 15 years. This retrospective overview summarises our results, describing the early and late clinical outcomes of 1,126 patients, including 370 ASD closures, 488 VSD closures and 268 valve surgeries, at a single center between October 2001 and December 2015. The mean follow-up time was 52 ± 35 months (range 8–120 months). The mean incision length was 6 ± 2.22 cm (range 3.9–8.9 cm). No patient required conversion to median sternotomy. All patients were satisfied with the cosmetic results at the follow-up assessment. No chest deformity or asymmetrical development of the breast was observed. Although there was no severe morbidity and operative mortality, ten late deaths occurred, 8 of which were due to cardiac causes and the other 2 to non-cardiac causes. RVIAT offers encouraging short- and long-term patient survival results and is a safe and reproducible approach with excellent late results. RVIAT should be considered as an alternative to conventional median sternotomy.

## Introduction

Minimally invasive approaches decrease the need for blood products and exhibit less morbidity and enhanced postoperative recovery^1,2^. Now the thoracoscope and robotic technology come to use in heart surgery, but limited by the local finance and long term training, the minimally invasive approaches are still the mainstream in China. Our center has performed right vertical infra-axillary thoracotomy (RVIAT) in valve surgery and congenital heart surgery for 15 years since October 2001, we have performed nearly 1,126 cases of RVIAT cardiac operations, and several reports have described surgical procedures using RVIAT^3–11^. We retrospectively analysed our experience in performing RVIAT and follow-up.

## Results

This retrospective overview summarises our experience and results with RVIAT among 1,126 selected patients with a mean age of 20 ± 15 years old (range 0.5–65 years). Of these patients, 379 (33.7%) were men, and 747 (66.3%) were women. The procedures consisted of 370 ASD closures, 488 VSD closures, and 268 valve surgeries over the past 15 years. No patient required conversion to median sternotomy. The mean patient body weight was 41 ± 19.73 kg (range 6–88 kg), and the BMI was 19 ± 3.96 kg/m^2^ (range 7.26–47.83 kg/m^2^). The mean incision length was 6 ± 2.22 cm (range 3.9–8 cm). All patients were satisfied with the postoperative analgesia.

### ASDs

There were included 370 ASD patients, 269 women (72.7%) and 101 men (27.3%) with a mean age of 23 ± 14 years (range 0.5–65 years). The mean diameter of defect was 2.67 ± 1.09 cm (range 0.2–6 cm). Direct suturing was used in 73 cases (19.7%) and a patch in 297cases (80.3%), while 52 patients underwent ASD repair combined with mitral valve repair, and 194 patients underwent ASD repair combined with tricuspid annuloplasty. As given in Table 1, the cardiopulmonary bypass time (CPB) durations in the arrested-heart group (340 patients) were shorter than in the beating-heart group (30 patients) (P < 0.01). The mean incision length was 4.4 ± 0.46 cm (range 3.9–5.2 cm). The mean total drainage volume was 137 ± 60.5 ml (range 0–387 ml), and 37 patients received blood transfusions (mean 303 ± 191.1 ml, range 100–750 ml).

**Table 1:** Preoperative and Operative Data of Patients with Congenital Heart Disease.

Variable		ASD n = 370	VSD n = 488
Body weight (kg, mean ± SD)		46 ± 17 (6–88)	32 ± 19 (6.5–88)
Height (cm, mean ± SD)		151 ± 23.40 (62–184)	128 ± 33 (62–190)
BMI (kg/m^2^, mean ± SD)		19 ± 4 (9.05–30.06)	18 ± 3.90 (7.26–47.83)
Concomitant defects, n			
Partial atrioventricular septal defect		9	18
Mitral insufficiency		88	46
Pulmonary artery hypertension		76	16
Tricuspid insufficiency		180	71
CPB (min, mean ± SD)	Arrested	*37* ± *17* (*n* *=* *340*)	*3*9 ± 18.*5* (*n* *=* *470*)
	Beating	*53* ± *16* (*n* *=* *30*)	*49* ± *19* (*n* *=* *18*)
Aortic clamp (min, mean ± SD)		*23* ± *13* (*n* *=* *340*)	*24* ± *15*.*5* (*n* *=* *470*)
ICU stay (days, mean ± SD)		3 ± 1	3 ± 1
Postoperative stay (days, mean ± SD)		10 ± 3	10 ± 3
Mechanical ventilation (hours, mean ± SD)		7 ± 4	7 ± 4
Additional procedures, n			
Partial atrioventricular septal defect closure		*9*	*18*
Mitral valve repair		*52*	*21*
Tricuspid annuloplasty		*194*	*66*

VSD, ventricular septal defect; ASD, atrial septal defect; SD, standard deviation; and BMI, body mass index; CPB, Cardiopulmonary bypass time.

### VSDs

There were included 488 VSD patients, 266 women (54.5%) and 222 men (45.5%) with a mean age of 12 ± 10 years (range 0.5–50 years). The mean diameter of the VSD defect was 0.97 ± 0.45 cm (range 0.2–5 cm). Direct suturing was used in 56 cases (11.5%) and a patch in 432 cases (88.5%), while 21 patients underwent VSD repair combined with mitral valve repair, and 66 patients underwent VSD repair combined with tricuspid annuloplasty. The mean incision length was 5.6 ± 0.8 cm (range 4.9–7.2 cm), the mean total drainage volume was 78 ± 66.3 ml (range 0–324 ml), and 47 patients received blood transfusions (mean 212 ± 143.8 ml, range 100–800 ml). As given in Table 1, the cardiopulmonary bypass time (CPB) durations in the arrested-heart group (470 patients) were shorter than in the beating-heart group (18 patients) (P < 0.01). TEE showed no increased aortic or semilunar valvular regurgitation after closure.

### Valve surgery

There were included 268 valve disease patients, 212 women (79.1%) and 56 men (20.9%), with a mean age of 41 ± 10 years (range 15–65 years). Most patients were classified as New York Heart Association (NYHA) functional Class II or III (112 patients each, 41.8%). The mean preoperative left ventricular ejection fraction was 0.575 ± 0.71 (range 0.33–0.71). The most predominant pathology was rheumatic valve disease (98.5%), followed by degeneration disease (0.75%) and infective endocarditis (0.75%).

The valve surgeries included 114 single and 114 double valve procedures (42.5% respectively) and 40 triple valve procedures (14.9%). Bioprosthetic valve was used in 116 cases and mechanical valve in 226 cases, while 44 patients combined with atrial fibrillation ablation, and 18 patients combined with left atrial thrombectomy. The CPB time was 127 ± 60 minutes (range 49–193 minutes), and the aortic cross-clamp time was 93 ± 25 minutes (range 27–151 minutes). The mean total drainage volume was 161 ± 87.9 ml (range 0–384 ml), and 88 patients received blood transfusions (mean 238 ± 123.5 ml, range 100–600 ml). The mean incision length was 7.3 ± 1.3 cm (range 5–8.9 cm). Concomitant procedures included 18 left atrial thrombectomies (6.7%), 44 atrial fibrillation ablations (16.4%), 2 left ventricular outflow tract reconstructions (0.7%) and 4 aortoplasties (1.5%). The results of the procedures are listed in Table 2.

**Table 2:** Preoperative and Operative Data of Valve Surgery Patients.

Variable	n = 268
**Body weight (kg, mean ± SD)**	58 ± 8.49 (41–84)
**Height (cm, mean ± SD)**	162 ± 6.2 (150–180)
**BMI (kg/m**^2^, **mean ± SD)**	27 ± 2.71 (16.00–30.82)
***Singel lesion, n***	**88**
*MS/MI/MS and MI*	*44/1*2*/16*
*AI/AS and AI*	*6/1*0
***Mixed lesions, n***	**180**
*two valves/three valves*	*124/56*
***Grade, n (%)***	
*grade + + *	*12* (*MS*)
*grade + + + *	*116* (*MS*) *+* *64*(*MI*) *+* *22*(*AS*) *+* *76*(*AI*)
*grade + + + + *	*82*(*MS*)*+6*0(*MI*)*+1*0(*AS*)*+4*0(*AI*)
**Procedure, n**	
***Single valve surgery***	***114***
*Mitral valve replacement/repair*	*84/12*
*aortic valve replacement*	18
***Double valves surgery***	***114***
*Mitral valve repair/replacement + tricuspid valve repair*	4/48
*Mitral valve repair/replacement + Aortic valve replacement*	6/56
***Triple valves surgery***	***4*** **0**
*Mitral valve replacement + aortic valve replacement/ repair + tricuspid valve repair*	34/4
*Mitral valve repair + aortic valve replacement + tricuspid valve repair*	*2*
**ICU stay (days, mean ± SD)**	4 ± 2
**Postoperative stay (days, mean ± SD)**	11 ± 3
**Mechanical ventilation (hours, mean ± SD)**	11 ± 4.4

SD, standard deviation; BMI, body mass index; MS, mitral valve stenosis; MI, mitral valve insufficiency; AI, aortic valve insufficiency; AS, aortic valve stenosis; and TI, tricuspid insufficiency; CPB, Cardiopulmonary bypass time; grade++, mitral valve area 1.0–1.5 cm^2^; grade+++, moderately insufficiency, or mitral valve area 1.0–1.5 cm^2^, or transvalvular pressure gradient 3.3–6.7 kpa (25–50 mmHg) in aortic stenosis; grade++++, severe insufficiency, or mitral valve area 1.5–2.0 cm^2^, or transvalvular pressure gradient >6.7 kpa (50 mmHg) in aortic stenosis.

### Follow-up assessment

**Figure 1:**
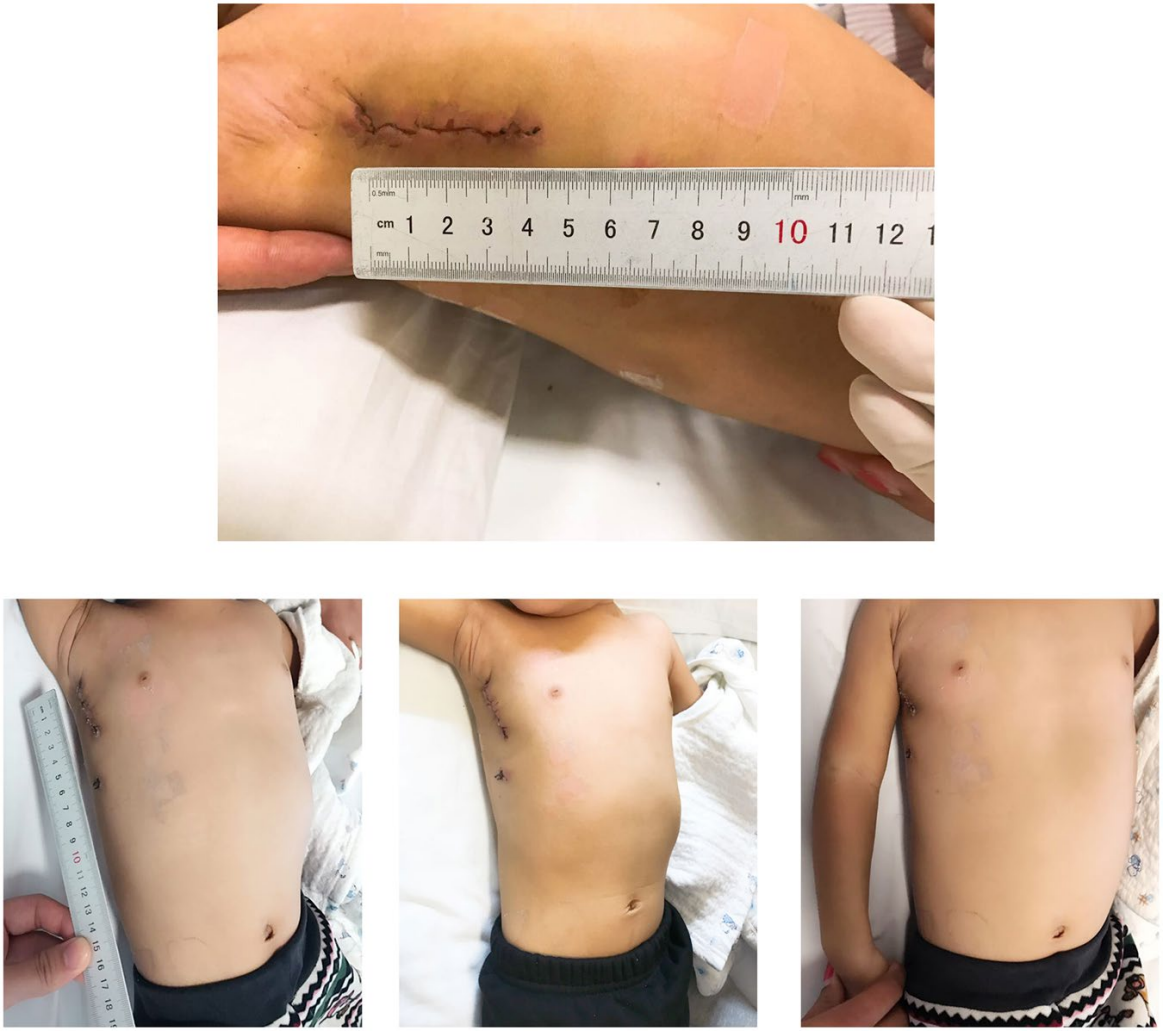
(Wang) Sutured incision after the operation. The skin incision along the right midaxillary line is not visible from a frontal view.

The follow-up period was 52 ± 35 months (range 8–120 months), and all the follow-up data were collected. No patient was lost to follow-up. The cosmetic advantage of RVIAT is a short incision, which is often invisible, under the armpit (Fig. 1(Wang)). All patients were satisfied with the cosmetic results at the follow-up assessment. No chest deformity or asymmetrical development of the breast was observed (Table 3).

**Table 3:** Postoperative Complications.

Atrial septal defects (n = 370)	
Postoperative morbidity, n (%)	7 (1.9)
Residual shunt	*0*
Reoperation for bleeding	*3* (*0*.*8*)
Incision complication	*1* (*0*.*3*)
Complete atrioventricular or right bundle-branch block	*0*
Transient sinus or supraventricular arrhythmia	*3* (*0*.*8*)
**Ventricular septal defects** (**n** **=** **488**)	
Postoperative morbidity, n (%)	6 (1.2)
Residual shunt	*4* (*0*.*8*)
Reoperation for bleeding	*1* (*0*.*2*)
Incision complication	*1* (*0*.*2*)
Complete atrioventricular or right bundle-branch block	*0*
Transient sinus or supraventricular arrhythmia	*0*
Second surgery for residual shunt	*0*
**Valve surgery** (**n** **=** **268**)	
Postoperative morbidity, n (%)	23 (8.6)
Re-exploration for bleeding	1 (0.4)
Incision complication	1 (0.4)
Stroke	0
Atrial fibrillation	21 (7.8)

### Early outcomes

#### ASD

During the follow-up period, the complication rate was 1.4% (n = 7 patients). Three patients (0.8%) required re-exploration for bleeding, and one patient (0.3%) presented with an incision complication. No cerebral strokes occurred in our series. Postoperative sinus bradycardia and atrial premature beats were observed in 3 patients (0.8%). These patients required medication for 3 to 5 days and recovered within one week. No episodes of residual ASD fistula or permanent rhythm disturbances occurred during the follow-up period.

#### VSD

During the follow-up period, the complication rate was 1.2% (n = 6 patients). One patient (0.2%) required a second operation for bleeding. Postoperative echocardiography revealed small residual shunts in 4 patients (0.8%), and one patient (0.2%) experienced an incision complication. All of the patients were in sinus rhythm. Two small residual shunts detected on echocardiography had healed at the 1-year follow-up visit. All patients were classified as having NYHA cardiac function Class I.

#### Valve surgery

During the follow-up period, the complications rate was 8.6% (n = 23 patients), including paroxysmal atrial fibrillation in 21 patients (7.8%), reoperation for bleeding in one patient (0.4%), and an incision complication in one patient (0.4%). No cerebral strokes occurred in our series.

### Late mortality and morbidity

Ten late deaths occurred, 8 of which were due to cardiac causes, and the other 2 were due to non-cardiac causes. No late deaths were observed in the ASD group, but 3 late deaths (cardiac causes) were observed in the VSD group, and 7 late deaths (5 cardiac causes and 2 non-cardiac causes) were observed in the valve surgery group.

## Discussion

The principal advantage of RVIAT is injury reduction. Firstly, RVIAT obviates the need to divide or incise muscles other than the intercostal muscles, and it preserves the long thoracic nerve and retains a normal space between the ribs and does not hinder the symmetrical development of the breasts and nipple sensitivity^12,13^. Secondly, as showed in Fig. 1(Wang), the limited cutaneous vertical incision under the armpit, providing better cosmetic results. Thirdly, the incision is in the chest wall which has low muscle mass and is far from the costochondral junction, it does not interfere with the development of the chest wall (Fig. 1(Wang)). In addition, femoral cannulation was not necessary^14,15^, it also avoids femoral artery injury.

The second advantage of RVIAT is easy to learn and practice compared with other minimally invasive approaches, thoracoscope or robotic technology^16^. RVIAT is mostly similar to the median sternotomy. RVIAT access can provide enough exposure of the ascending aorta and both venae cavae as well as improve the visualisation of the right atrium and valves, which can be examined under direct vision. The use of a straight arterial cannula, the procedure, and cannulation are similar to the median sternotomy. There is a little difficult to close subpulmonary VSDs through a right atriotomy, however, we found that a right ventricular approach can often provide acceptable vision when a wet sponge is placed in the pericardial cavity beneath the heart^4,5^.

The third advantage of RVIAT is it does not require special surgical instruments and does not increase costs. And the entire RVIAT procedure is performed under direct vision without additional devices, such as video-assisted thoracoscopic equipment. RVIAT may be one of the most economical and practical procedure compared with other minimally invasive approaches, the thoracoscope and robotic technology.

We repaired ASD and VSD on beating-heart without cross-clamping the aorta (Table 1). Several reports have described this beating-heart techniques^3,17^. The advantages of this techniques include avoidance of ischaemia–reperfusion injury, less use of inotropic medications, and additionally less emboli produceing during cross-clamping. To prevent air embolism, we insufflated the chest with carbon dioxide during the procedure. Any air in the left atrium would be carbon dioxide, which is easily absorbed. We also use transesophageal echocardiography to check whether there is any air stay in atrium or ventricle. In this study, there is no embolic complication after surgery. ASD and VSD repair on a beating heart is an effective and safe alternative to techniques.

We also found that a BMI greater than 30 kg/m^2^, age or the heart function (Tables 1, 2) was not the contraindication for this procedure. More and more elder patients prefer to choose the minimally invasive for a quick recovery in china. RVIAT is associated with significantly fewer blood transfusions and less chest drainage, most likely because of the avoidance of a sternotomy, which might contribute to the quick recovery. In addition, as show in our follow, the use of RVIAT did not increase the incidence of early complications, and the visual analogue scale score used to measure postoperative pain did not indicate that the spreading of the ribs was more painful than a sternotomy^18,19^. Furthermore, RVIAT did not influence the results of the coughing exercise after surgery, which was beneficial for early rehabilitation.

Our center have rich experiment in RVIAT for more than one thousand patients with ASD, VSD and valve diseases, it demonstrated to be safe and a cosmetically better alternative to median sternotomy according to our other reports. RVIAT can avoid the potential disadvantages of other minimally invasive approaches, including femoral artery cannulation and its complications, the extra expense of special surgical instrumentation, sternum infection associated with standard sternotomy, and injury to the mammary gland. The RVIAT procedure is familiar to cardiac surgeons, and it is easy to grasp after experience with correcting relatively simple cardiac defects^20,21^. RVIAT has advantages, and our follow-up assessment suggests that this approach will not cause breast or pectoral muscle maldevelopment. In addition, the operative scar is less evident.

A limitation of this study is not possible to perform multivariate analysis to identify predictors for mobility and mortality because the numbers were too low. And we have not found any facts such as age, sex or others contribute to the follow-up results (P > 0.05). This study was based on the retrospective information from our center database and some variables used in this study, such as length of intensive care unit stay and hospital stay, were determined largely by the speed of the patient’s recovery from surgery, which can be influenced by subjective factors. In addition, a postoperative assessment of the quality of life was not performed. And the comparison between RVIAT and the median thoracotomy or interventional treatment is also being undertaken.

In conclusion, this group of patients could be the largest group reported to date for RVIAT, and the medium and long-term results were satisfactory.

## Methods

The institutional review board of Nanjing University approved the present study, which was in compliance with the Declaration of Helsinki. The institutional review board waived the need for individual patient consent.

We retrospectively reviewed the data of 1,126 consecutive patients who underwent valve or congenital heart surgery at our institution between January 2001 and December 2015. We sought to evaluate the outcomes of patients who underwent valve surgery and congenital heart surgery (ASD or VSD) via the RVIAT approach. All of the patients received a preoperative transthoracic echocardiography on admission and at discharge. The severity of valve regurgitation/stenosis was graded according to the recommendations of the European Society of Cardiology and the European Association for Cardiothoracic Surgery.

### Clinical trial registry and IRB approval

This is an observational study; it was approved by the institutional review board of Nanjing University and in compliance with the Declaration of Helsinki. The institutional review board waived the need for individual patient consent.

### Minimally invasive surgery via RVIAT

Our RVIAT approach was previously described^3–12^. After general anaesthesia, patients were intubated with a double-lumen tube. A transoesophageal echocardiography (TEE) probe was inserted.

Briefly, the operation was generally performed through the second or third intercostal space to the fifth intercostal space along the right midaxillary line. No rib was fractured using this approach. Cannulation of the ascending aorta, inferior vena cava, and superior vena cava was performed^4–12^. A 2-cm incision was made in the seventh intercostal space for inferior vena cava cannulation for better exposure and chest drainage postoperatively. Only subpulmonary VSDs were closed through an approximately 2- to 3-cm incision of the right ventricular outflow tract 4. Application of an analgesia pump is able to relieve pain after the operation.

### Follow-up period

The study cohort was composed of 1,126 patients. Early mortality was attributed to operative mortality, which was defined as any death occurring within 30 days of the operation or before hospital discharge; long-term survival was also evaluated. Long-term outcomes were determined from clinical records when available or by direct patient contact via telephone interviews when necessary. All of the follow-up data were collected, and no patient was lost to follow-up. The data were collected until August 2016.

### Statistical analyses

Descriptive statistics are reported as the means ± standard deviations (SDs) for continuous variables and as frequencies and percentages for categorical variables. Statistical significance was determined using Student’s t-test for continuous variables and the χ^2^ analysis for categorical variables. All tests were considered as statistically significant at P < 0.05. All analyses were performed using SPSS Statistics Version 22.0 (SPSS Inc., Chicago, IL, USA).
